# Compromised Lung Volume and Hemostatic Abnormalities in COVID-19 Pneumonia: Results from an Observational Study on 510 Consecutive Patients

**DOI:** 10.3390/jcm10132894

**Published:** 2021-06-29

**Authors:** Ezio Lanza, Maria Elisa Mancuso, Gaia Messana, Paola Ferrazzi, Costanza Lisi, Pierpaolo Di Micco, Stefano Barco, Luca Balzarini, Corrado Lodigiani

**Affiliations:** 1 Department of Radiology, IRCCS Humanitas Research Hospital, 20089 Milan, Italy; ezio.lanza@humanitas.it (E.L.); luca.balzarini@humanitas.it (L.B.); 2 Center for Thrombosis and Hemorrhagic Diseases, IRCCS Humanitas Research Hospital, 20089 Milan, Italy; paola.ferrazzi@humanitas.it (P.F.); corrado.lodigiani@humanitas.it (C.L.); 3 Department of Biomedical Sciences, Humanitas University, 20090 Milan, Italy; gaia.messana@gmail.com (G.M.); costanza.lisi95@gmail.com (C.L.); 4 Department of Internal Medicine, Ospedale Fatebenefratelli, 80123 Naples, Italy; pdimicco@libero.it; 5 Center for Thrombosis and Hemostasis, University Medical Center, Johannes Gutenberg University Mainz, 55122 Mainz, Germany; stefano.barco@usz.ch; 6 Clinic for Angiology, University Hospital Zurich, 8091 Zurich, Switzerland

**Keywords:** COVID-19, lung volume measurements, D-dimer, hemostasis, SARS-CoV-2 pneumonia

## Abstract

Background: Hemostatic abnormalities have been described in COVID-19, and pulmonary microthrombosis was consistently found at autopsy with concomitant severe lung damage. Methods: This is a retrospective observational cross-sectional study including consecutive patients with COVID-19 pneumonia who underwent unenhanced chest CT upon admittance at the emergency room (ER) in one large academic hospital. QCT was used for the calculation of compromised lung volume (%CL). Clinical data were retrieved from patients’ files. Laboratory data were obtained upon presentation at the ER. Aim: The aim of this study was to evaluate the correlation between hemostatic abnormalities and lung involvement in patients affected by COVID-19 pneumonia as described using computer-aided quantitative evaluation of chest CT (quantitative CT (QCT)). Results: A total of 510 consecutive patients (68% males), aged 67 years in median, diagnosed with COVID-19 pneumonia, who underwent unenhanced CT scan upon admission to the ER, were included. In all, 115 patients had %CL > 23%; compared to those with %CL < 23%, they showed higher levels of D-dimer, fibrinogen, and CRP, greater platelet count, and longer PT ratio. Via multivariate regression analysis, BMI ≥ 30 kg/m^2^, D-dimer levels > 500 ng/mL, CRP > 5.0 ng/mL and PT ratio > 1.2 were found to be independent predictors of a %CL > 23% (adjusted odds ratios (95% confidence intervals): 2.1 (1.1–4.0), 3.1 (1.6–5.8), 2.4 (1.3–4.5), and 3.4 (1.4–8.5), respectively). Conclusions: Hemostatic abnormalities in patients affected by COVID-19 correlate with the severity of lung injury as measured by %CL. Our results underline the pathogenetic role of hemostasis in COVID-19 pneumonia beyond the presence of clinically evident thromboembolic complications.

## 1. Introduction

Coronavirus disease 2019 (COVID-19) is a novel, potentially fatal respiratory infection caused by coronavirus SARS-CoV-2, which was first identified in December 2019 in Wuhan, Hubei, China [1], and has since spread globally. Europe and the United States of America have been the epicenters of COVID-19, with a total of 36,607,500 and 50,612,223 confirmed cases and a total of 830,948 and 1,196,316 deaths reported as of 25 February 2021, respectively [2].

As the pandemic progressed, the scientific community accelerated the study of predictors of an unfavorable course, as measured by hospitalization time, need for invasive ventilation, and the risk of death [3]. An interesting area of knowledge pertains to the association between hemostatic abnormalities and thrombotic events complicating COVID-19 pneumonia [4,5]. D-dimer levels were found to be increased in the vast majority of patients with COVID-19 and represent the main predictor of coagulation-associated complications, as well as death [6,7]. Together with C-reactive protein, platelet count and erythrocyte sedimentation rate have been reported as the main predictors for thrombotic events during hospitalization [8]. Indeed, contrast-enhanced CT angiography has unveiled a high prevalence of acute pulmonary embolism (PE) in severe cases [9,10]. Moreover, the use of computer-aided quantitative analysis (quantitative computed tomography (QCT)), measuring the volume of well-aerated and compromised lungs, has also been proven to be a potential predictor of prognosis [11].

In the present analysis, we used QCT to evaluate the severity of compromised lung volume (%CL) in a large cohort of patients admitted to an academic hospital and investigated the potential association between %CL and hemostatic abnormalities occurring during COVID-19 infection.

## 2. Materials and Methods

### 2.1. Subjects

Eligible patients were consecutive patients admitted to the emergency room (ER) of a large academic hospital in Milan with COVID-19 pneumonia and who underwent unenhanced chest CT between February and April 2020. Unenhanced chest CT was performed upon ER admittance in all patients in concomitance with real-time polymerase chain reaction (RT-PCR) for SARS-CoV-2.

### 2.2. Study Design

This study was a retrospective, observational, cross-sectional study conducted in accordance with the amended Declaration of Helsinki. The Institutional Review Board of IRCCS Humanitas Research Hospital approved the protocol, and written informed consent was obtained from all patients before every CT exam.

Medical records of patients diagnosed with COVID-19 by means of real-time reverse transcription–polymerase chain reaction (RT-PCR) were reviewed. Inclusion criteria for this study were (a) unenhanced chest CT performed upon admission to the ER and (b) COVID-19 pneumonia. Demographic and clinical data were collected for all patients, including laboratory parameters. Cardiovascular disease (CVD) was defined as known coronary artery disease, previous stroke/TIA and peripheral artery disease, and/or carotid artery stenosis. Chronic kidney disease (CKD) was defined as a value of filtration rate < 60 mL/min calculated using CKD-EPI. PE was searched by CT pulmonary angiography in subjects with an unexplained clinical worsening of respiratory function or a rapid increase in D-dimer levels (D-Dimer HS, Instrumental Laboratory Company, Bedford, MA, USA). The International Society on Thrombosis and Haemostasis (ISTH) score for overt disseminated intravascular coagulation (DIC) was calculated [12], and DIC was considered present with a score ≥5. Deaths were also reported.

### 2.3. Quantitative CT Analysis

All CT scans were performed on a 64-row scanner (Ingenuity, Philips, Amsterdam, The Netherlands) dedicated to COVID-19 and isolated from the rest of the radiology department, using standard parameters for chest imaging (100–140 kV, mAs according to size, slice thickness = 2 mm). DICOM images were exported to 3D-Slicer (www.slicer.org; last accessed 26 May 2021), which is open-source software for image segmentation and quantification. The software performed a first-pass, semi-automated segmentation of the lung parenchyma; missing lung volumes were manually outlined using tools such as spherical brushes and three-dimensional erasers. A complete segmentation included both lungs with interstitial structures, segmentary vessels, and bronchi; the main pulmonary arteries and bronchi, all mediastinal structures, pleural effusions, and lung masses were excluded. Contrast-enhanced scans, although available in some cases, were not used for segmentation purposes as the presence of a contrast medium would have altered the density of the lung parenchyma.

The whole lung parenchyma was divided into different volume subunits, according to Hounsfield Units (HU) intervals validated in previous research [13], and categorized into non-aerated (%NNL, 100, −100 HU), poorly aerated (%PAL, −500, −100 HU), normally aerated (%NAL, −900, −501 HU), and hyper-inflated (%HIL, −1000, −900 HU). The sum of %NAL and %HIL defined the normal lung volume, while the sum of %PAL and %NNL defined the compromised lung volume (%CL, −500, 100 HU) that represents the main outcome of this study.

### 2.4. Outcome

The primary objective of this study was to evaluate the correlation between hemostatic abnormalities and lung damage in patients affected by COVID-19 pneumonia as described using computer-aided quantitative evaluation of chest CT (quantitative CT (QCT)).

### 2.5. Statistical Analysis

Continuous variables, expressed as median values and interquartile ranges (IQR), were compared using the Mann–Whitney U-test. Categorical variables, expressed as frequencies and percentage values, were compared via the chi-squared test. Correlation coefficients were calculated using Spearman’s rho tests. %CL > 23% has been considered the primary outcome because it has been previously related to the worse respiratory outcome [14]. A BMI ≥ 30 kg/m^2^, a D-dimer value > 500 ng/mL, fibrinogen levels > 550 mg/dL, PT ratio > 1.20, PLTs count > 200 × 10^3^/mmc, and CRP values > 5 mg/dL were considered putative risk factors for having a %CL > 23%. Cut-off values for BMI were chosen to identify obese patients; those for D-dimer and CRP were based on published literature [15,16], those of fibrinogen and PLT count were based on the median values observed in the study population, and those for PT were based on the normal laboratory range for this test. Odds ratios (OR) with 95% confidence intervals (95%CI) were calculated for all the aforementioned variables via univariate logistic regression analysis (crude OR); all variables with significant 95%CI were then included in the multivariate model to adjust for confounders and identify independent predictors of having a %CL > 23% (adjusted OR, aOR). No missing data imputation was used, and multivariate analysis was performed including only subjects with complete data. All reported *p* values are two-sided, and values <0.05 were considered significant. All analyses were performed using IBM SPSS Statistics software (version 26.0, IBM Corp., Armonk, New York, NY, USA).

## 3. Results

In this study, we included 510 consecutive patients (347 males, 68%) aged in median 67 years (interquartile range (IQR), 56–77), who were diagnosed with COVID-19 pneumonia and underwent unenhanced CT scan upon admission to the ER. The main characteristics of these subjects are summarized in Table 1.

In all, 384 patients (75%; median age: 62 years, IQR: 53–71) successfully recovered and were discharged from hospital. A total of 126 (25%; median age 77 years, IQR: 72–84), died during hospitalization.

Laboratory and clinical parameters upon admission (median, IQR) in survivors and non-survivors are shown in Table 2. Deceased patients had higher D-dimer and CRP levels, prolonged PT ratio, higher WBC count, and greater %CL compared to survivors (Table 2).

During the hospital stay, 402 (79%) patients required oxygenation support, this being via a nasal cannula in 143 (36%), facial mask in 31 (8%), Venturi mask in 84 (21%), helmet CPAP in 66 (16%), and intubation in 78 (19%). Overall, 86 patients (17%) required ICU hospitalization, PE was found in 11 patients (2.2%), and DIC occurred in 7 patients (1.4%).

### 3.1. Quantitative CT Analysis

**Figure 1:**
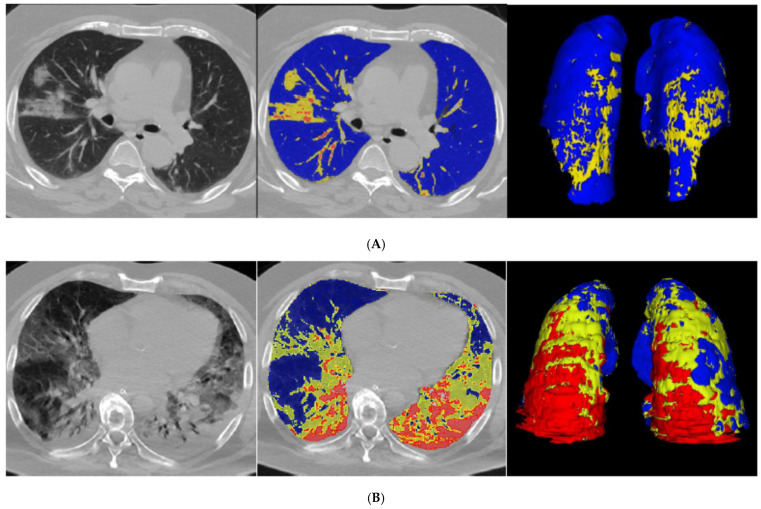

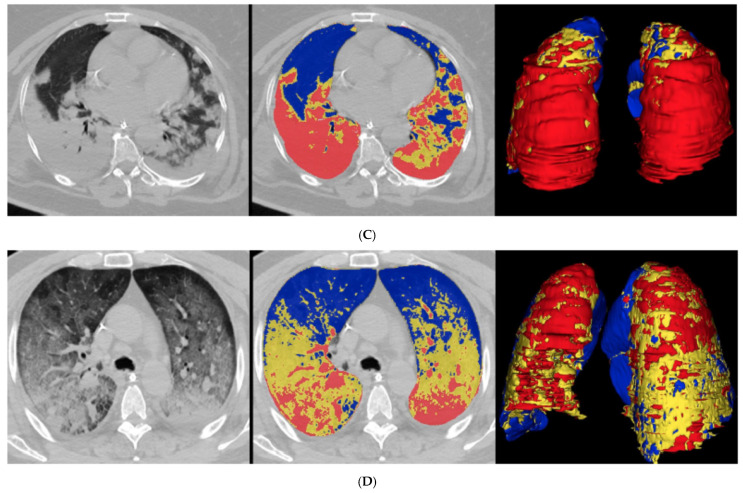
Unenhanced CT scan and QCT analysis in four patients with COVID-19 pneumonia. (**A**,**B**) show one QCT with %CL < 23% and one with %CL > 23%. (**C**,**D**) highlight the texture differences between the “poorly aerated” volume (%PAL, −500, −100 HU) and “non-aerated” volume (%NNL, 100, -100 HU). (**A**) refers to a 71-year-old man, oxygenated through nasal cannulae. Upon admission, laboratory tests were as follows: D-dimer 199 ng/mL, fibrinogen 416 mg/dL, PT ratio 1.03, CRP 1.0 mg/dL. %CL was 10%. Non-contrast axial chest CT showed ground-glass opacities, predominantly in the middle lobe; quantitative analysis highlights poorly aerated lung volumes in yellow (8% PAL) and normally aerated lung volumes in blue. (**B**) refers to a 64-year-old man who required mechanical ventilation and intubation. Upon admission, laboratory tests were as follows: D-dimer 1161 ng/mL, fibrinogen 1021 mg/dL, PT ratio 1.18, CRP 45.3 mg/dL. %CL was 55%. Non-contrast axial chest CT showed diffuse bilateral ground-glass opacities and posterior lung consolidation; quantitative analysis highlights non-aerated lung volumes in red (19% NNL), poorly aerated lung volumes in yellow (36% PAL), and normally aerated lung volumes in blue. (**C**) refers to a 65-year-old woman who required mechanical ventilation and intubation. Upon admission, laboratory tests were as follows: PT ratio 1.34, CRP 31.9 mg/dL. %CL was 66%. Non-contrast axial chest CT showed extensive posterior consolidation of the right lower lung lobe and patchy dense consolidation in the subpleural space of both left lower and upper lung lobes; quantitative analysis highlights poorly aerated lung volumes in yellow (23% PAL) and non-aerated lung volumes in red (43% NNL). (**D**) refers to a 66-year-old man who required mechanical ventilation and intubation. Upon admission, laboratory tests were as follows: D-dimer 1335 ng/mL, fibrinogen 832 mg/dL, PT ratio 1.20, CRP 33.8 mg/dL. %CL was 62%. Non-contrast axial chest CT showed extensive ground-glass consolidation with “crazy paving” pattern typical of COVID-19; quantitative analysis highlights normally aerated lung volumes in blue, non-aerated lung volumes in red (12% NNL), and poorly aerated lung volumes in yellow (50% PAL).

All CT scans were of satisfactory quality for analysis. Case examples of QCT with %CL < or >23% and different texture between %PAL and %NNL are illustrated in Figure 1. Median %CL was 16% in the deceased group (IQR, 10–32%) and 11% in the recovered group (IQR, 6–19%). The difference proved statistically significant (*p* < 0.001; Table 2).

A total of 115 had %CL > 23%. Compared to those with %CL< 23%, they showed higher levels of D-dimer, fibrinogen, and CRP, greater platelet count, and longer PT ratio (Table 3). A greater proportion of these patients had BMI ≥ 30 kg/m^2^ (35% vs 22%, *p* = 0.007). No significant difference was found with respect to sex, age, or comorbidities (Table 3). The need for any type of oxygenation support and the proportion of mechanical ventilation, as well as mortality, were greater in this group of patients (*p* < 0.001 for both outcomes; Table 3).

Among those 11 patients subsequently diagnosed with PE, only 6 (54%) displayed %CL > 23%.

**Figure 2:**
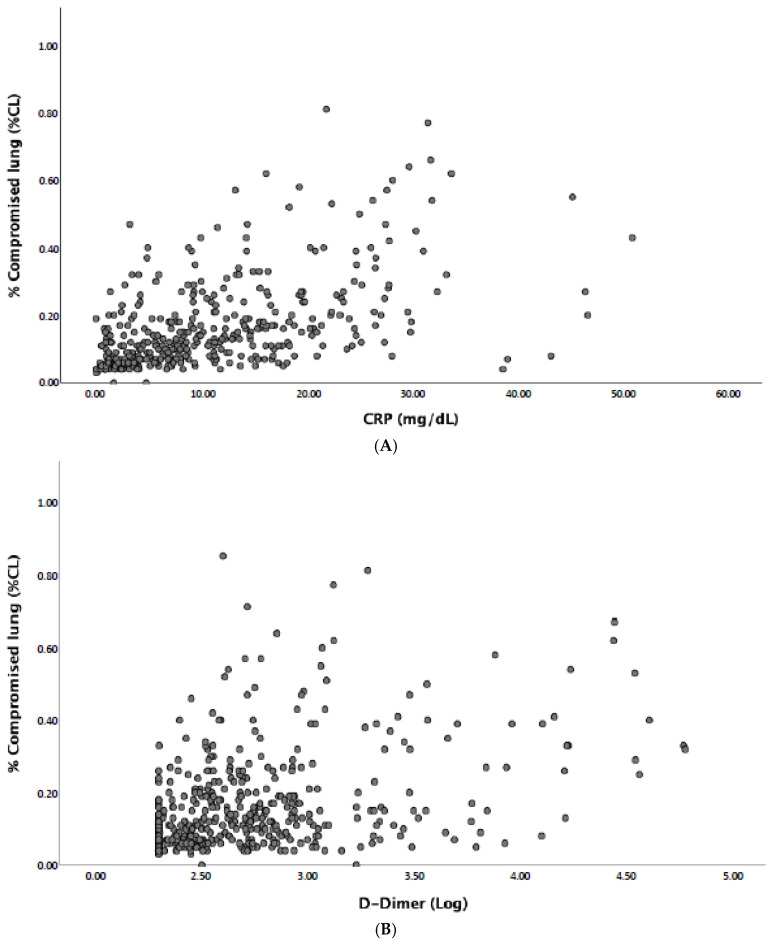
Scatter plot of the distribution of CRP ((**A**); r^2^ = 0.58, *p* < 0.001) and log D-dimer ((**B**); r^2^ = 0.36, *p* < 0.001) levels according to %CL values in the study population.

A significant correlation was found between %CL and CRP (r^2^ = 0.58, *p* < 0.001), D-dimer (r^2^ = 0.36, *p* < 0.001), fibrinogen (r^2^ = 0.33, *p* < 0.001), WBC count (r^2^ = 0.31, *p* < 0.001), PT ratio (r^2^ = 0.28, *p* < 0.001), platelet count (r^2^ = 0.22, *p* < 0.001), and age (r^2^ = 0.12, *p* = 0.005). Figure 2 shows a scatter plot of RCP (A) and D-dimer (B) values in correlation with %CL value distribution in the study population.

### 3.2. Hemostatic Abnormalities and Compromised Lung

The results of univariate and multivariate logistic regression analysis of potential determinants of %CL are shown in Table 4. Multivariate analysis was run in 287 patients with complete clinical and laboratory data. After adjustment, BMI ≥ 30 kg/m^2^, D-dimer levels > 500 ng/mL, CRP > 5.0 ng/mL, and PT ratio > 1.2 were found to be independent predictors of a %CL > 23%.

## 4. Discussion

In this study, we found that elevated D-dimer levels and prolonged PT values upon admission in patients affected by COVID-19 pneumonia were independent predictors of the extent of lung impairment as measured by means of QCT.

Chest CT is the standard imaging technique to identify COVID-19 pneumonia, but it is limited to visual findings; by contrast, QCT analysis adds knowledge on functional lung impairment, which is strictly related to the management and prognosis of these patients. In fact, in a series of 222 patients, compromised lung volume proved to be the most accurate predictor of the need for respiratory support, with a value >23% meaning being at high risk for intubation [14]. Similarly, in our series, patients with a %CL > 23% displayed a greater need for mechanical ventilation as well as increased mortality.

The usefulness of quantifying the volume of the compromised lung from a simple unenhanced chest CT performed upon admission resides in the fact that this method can identify minimal changes in voxel units that may not be visible to the unaided eye. Many studies have reported its use in research on interstitial lung diseases, pulmonary sarcoidosis, and pulmonary vascular diseases [17,18,19].

The association between alterations of hemostasis and infections is well known [20]. Elevated levels of D-dimer and fibrin degradation products (FDP) characterize the initial coagulopathy of COVID-19 pneumonia and have been associated with disease severity and survival [15,21,22,23,24]. Several studies have been conducted to demonstrate a relation between coagulation parameter derangement and patient outcome, both in terms of ICU admission and mortality. Indeed, among 183 COVID-19-affected Chinese patients, non-survivors revealed significantly higher D-dimer and FDP levels, prolonged prothrombin time, and activated partial thromboplastin time at admission compared to survivors [25]. More recently, in 67 non-Chinese COVID-19 patients, it was first shown that clotting alterations, with a trend toward a hypercoagulable state due to increased values of D-dimer and fibrinogen, were present at the early stage of the disease and are more likely associated with a more severe form of COVID-19, characterized by severe acute respiratory syndrome (SARS) [26].

In our study, which includes more than 500 consecutive patients evaluated upon admission to the ER, we confirm that non-survivors had elevated D-dimer levels and prolonged PT ratio, with CRP also being significantly higher in this group of patients. All these three laboratory markers were found to be independently associated with functional lung impairment, highlighting that coagulation alterations seen in COVID-19 are strictly related to the inflammatory response triggered by acute viral infection and that a concerted action of coagulation and inflammation might be related to the observed lung injury. Indeed, in a Chinese study including 147 patients, coagulation abnormalities were prominent in ICU patients, and prolonged PT, elevated FDP, and D-dimer were linearly correlated to increased levels of IL2R, IL6, IL8, and TNF-α [22].

The hypothesis that a continuous crosstalk between hemostasis and inflammation influences the severity of lung impairment in COVID-19 patients is suggested by the fact that pneumocytes and endothelial cells are primary targets of SARS-CoV2 infection [27] and that alveolar damage and microvascular thrombosis are the main postmortem histopathologic findings [28]. QCT analysis might reflect the extent of lung injury related to those physiopathological mechanisms beyond the presence of thromboembolic complications. In fact, in our cohort, PE was found in a minority, and %CL >23% was found in only half of them.

Some authors recently hypothesized that pulmonary “thrombosis” rather than “embolism” may be involved in COVID-19 pneumonia [29]; this is also suggested by preliminary data from retrospective reports on the use of dual-energy computed tomography (DECT) [30,31]. In these studies, DECT imaging also demonstrated pulmonary ischemic areas in the absence of PE, which could be the consequence of the microvascular lesions caused by COVID-19 [30,31]. Standardized QCT as presented in our study may have a similar value to that of DECT, showing patterns of lung function impairment related to the same microvascular lesions without the need for intravenous contrast injection, and going beyond the limitations of a subjective radiological interpretation.

Our study has certain limitations: It is a retrospective study; imaging for PE was not performed in all patients but only in those with clinical suspicion, leading to a possible underestimation of this feature in our patient population; and there is no histological evidence of a greater extent/proportion of microvascular lung thrombosis in patients with greater %CL.

In conclusion, hemostatic abnormalities in patients affected with COVID-19 correlate with the severity of lung injury as measured by compromised lung volume, a parameter derived by computer-aided quantitative analysis of non-contrast chest CT that represents a measure of pulmonary dysfunction as well as a risk factor for a more severe respiratory outcome and in-hospital mortality. Our results underline the pathogenetic role of hemostasis in COVID-19 pneumonia beyond the presence of clinically evident thromboembolic complications.

## Figures and Tables

**Table 1:** Main characteristics of the 510 consecutive patients admitted to the emergency room with COVID-19 pneumonia included in the study.

Characteristics	Patients (*n* = 510)
Median age, years (IQR)	67 (56–77)
Male sex (%)	347 (68)
BMI ≥ 30 kg/m^2^ (%)	119/478 (25)
Smokers (%)	58/509 (11)
Diabetes (%)	117/508 (23)
Hypertension (%)	251/508 (49)
Cardiovascular disease (%)	121 (24)
COPD (%)	46/508 (9)
CKD (%)	81/508 (16)
Cancer (%)	34/505 (7)
Median %CL (IQR)	12 (7–21)
Median platelet count, ×10^3^/mmc (IQR)	202 (154–267)
Median WBC count, ×10^3^/mmc (IQR)	6.7 (5.2–9.7)
Median D-dimer levels, ng/mL (IQR)	434 (267.2–842.5)
Median fibrinogen levels, mg/dL (IQR)	556 (450–676)
Median prothrombin time ratio (IQR)	1.13 (1.06–1.23)
Median activated partial thromboplastin time ratio (IQR)	0.97 (0.90–1.06)
Median CRP, mg/dL (IQR)	8.8 (3.7–16.2)

BMI = body mass index; COPD = chronic obstructive pulmonary disease; CKD = chronic kidney disease; CL = compromised lung; WBC = white blood cells; CRP = C-reactive protein. Normal values for laboratory parameters: platelets 150–400 × 10^3^/mmc, WBC 4–10 × 10^3^/mmc, D-dimer 200–350 ng/mL, fibrinogen 160–400 mg/dL, PT ratio 0.9–1.2, aPTT ratio 0.8–1.2, CRP < 1 mg/dL.

**Table 2:** Clinical and laboratory features of 510 consecutive patients with COVID-19 pneumonia included in the study and compared according to survival outcome.

Characteristics	Survivors (*n* = 384)	Non-Survivors (*n* = 126)	*p* Value
Median age, years (IQR)	62 (53–71)	77 (72–84)	<0.001
Male sex (%)	262 (68)	85 (67)	0.91
BMI ≥ 30 kg/m^2^ (%)	94/371 (25)	25/107 (23)	0.71
Smokers (%)	37/383 (10)	21 (17)	0.05
Diabetes (%)	73/383 (19)	44/125 (35)	<0.001
Hypertension (%)	164/383 (43)	87/125 (70)	<0.001
Cardiovascular disease (%)	72 (19)	49 (39)	<0.001
COPD (%)	30/383 (8)	16/125 (13)	0.11
CKD (%)	38/383 (10)	43/125 (34)	<0.001
Cancer (%)	16/380 (4)	18/125 (14)	<0.001
Median %CL (IQR)	11 (6–19)	16 (10-32)	<0.001
Median D-dimer levels, ng/mL (IQR)	365 (243–662)	734 (383–2238)	<0.001
Median fibrinogen levels, mg/dL (IQR)	552 (454–668)	564 (449–702)	0.61
Median PT ratio (IQR)	1.12 (1.06–1.20)	1.19 (1.08–1.34)	<0.001
Median aPTT ratio (IQR)	0.97 (0.91–1.05)	0.97 (0.89–1.09)	0.88
Median platelet count, ×10^3^/mmc (IQR)	204 (163–264)	200 (130–272)	0.09
Median CRP, mg/dL (IQR)	7.7 (3.1–14.4)	13.3 (7.5–21.5)	<0.001
Median WBC count, × 10^3^/mmc (IQR)	6.5 (5.2–8.9)	7.9 (5.4–11.9)	0.01

BMI = body mass index; COPD = chronic obstructive pulmonary disease; CKD = chronic kidney disease; CL = compromised lung; PT = prothrombin time; aPTT = activated partial thromboplastin time; CRP = C-reactive protein; WBC = white blood cells. Normal values for laboratory parameters: platelets 150–400 × 10^3^/mmc, WBC 4–10 × 10^3^/mmc, D-dimer 200–350 ng/mL, fibrinogen 160–400 mg/dL, PT ratio 0.9–1.2, aPTT ratio 0.8–1.2, CRP < 1 mg/dL.

**Table 3:** Clinical and laboratory features of 115 patients with COVID-19 pneumonia and >23% of compromised lung as measured by QTC and comparison with 395 patients with COVID-19 pneumonia and <23% of compromised lung at QTC.

Characteristics	Patients with %CL >23% (*n* = 115)	Patients with %CL <23% (*n* = 395)	*p* Value
Median age, years (IQR)	68 (58–75)	67 (55–77)	0.43
Male sex (%)	79 (69)	268 (68)	0.91
BMI ≥ 30 kg/m^2^ (%)	37/105 (35)	82/373 (22)	0.007
Smokers (%)	11/114 (10)	47 (12)	0.62
Diabetes (%)	27/113 (24)	90 (23)	0.80
Hypertension (%)	55/113 (49)	196 (50)	0.92
Cardiovascular disease (%)	26 (23)	95 (24)	0.80
COPD (%)	6/113 (5)	40 (10)	0.14
CKD (%)	22/113 (20)	59 (15)	0.25
Cancer (%)	5/112 (5)	29/395 (7)	0.39
Median %CL (IQR)	33 (27-43)	10 (6-14)	<0.001
Median D-dimer levels, ng/mL (IQR)	704 (405–2777)	358 (242–677)	<0.001
Median fibrinogen levels, mg/dL (IQR)	639 (515–778)	537 (437–634)	<0.001
Median PT ratio (IQR)	1.19 (1.11–1.33)	1.11 (1.06–1.20)	<0.001
Median aPTT ratio (IQR)	0.97 (0.87–1.07)	0.97 (0.91–1.05)	0.22
Median platelet count, ×10^3^/mmc (IQR)	246 (177–344)	196 (148–246)	<0.001
Median CRP, mg/dL (IQR)	17.4 (10.1–26.2)	7.2 (2.8–13.1)	<0.001
Median WBC count, ×10^3^/mmc (IQR)	8.7 (6.4–12.2)	6.1 (5.0–8.7)	<0.001
Need for oxygenation support (%)	115 (100)	287 (73)	<0.001
Mechanical ventilation (%)	49/115 (43)	29/287 (10)	<0.001
Non-survivors (%)	46 (40)	80 (20)	<0.001

QCT = quantitative computed tomography; BMI = body m index; COPD = chronic obstructive pulmonary disease; CKD = chronic kidney disease; CL = compromised lung; PT = prothrombin time; aPTT = activated partial thromboplastin time; CRP = C-reactive protein; WBC = white blood cells. Normal values for laboratory parameters: platelets 150–400 × 10^3^/mmc, WBC 4–10 × 10^3^/mmc, D-dimer 200–350 ng/mL, fibrinogen 160–400 mg/dL, PT ratio 0.9–1.2, aPTT ratio 0.8–1.2, CRP < 1 mg/dL.

**Table 4:** Univariate and multivariate logistic regression analysis of potential predictors of %CL > 23% in 287 patients with complete clinical and laboratory data included in the study.

Variable	OR (95% CI)	adjOR (95% CI)
BMI ≥ 30 kg/m^2^	1.9 (1.2–3.1)	2.1 (1.1–4.0)
D-dimer levels > 500 ng/mL	2.7 (1.7–4.3)	3.1 (1.6–5.8)
Fibrinogen levels > 400 mg/dL	2.1 (1.0–4.4)	1.5 (0.5–4.3)
PT ratio > 1.20	2.6 (1.7–4.1)	2.4 (1.3–4.5)
Platelet count > 200 × 10^3^/mmc	2.3 (1.5–3.6)	1.9 (0.9–3.5)
CRP > 5 mg/dL	5.6 (2.7–11.5)	3.4 (1.4–8.5)

OR = odds ratio; CI = confidence interval; BMI = body mass index; CL = compromised lung; PT = prothrombin time; CRP = C-reactive protein.

## Data Availability

The data presented in this study are available on request from the corresponding author. The data are not publicly available due to privacy.
